# Disturbance intensification is altering the trait composition of Caribbean reefs, locking them into a low functioning state

**DOI:** 10.1038/s41598-023-40672-x

**Published:** 2023-08-28

**Authors:** Laura Mudge, John F. Bruno

**Affiliations:** 1 Department of Biology, University of North Carolina at Chapel Hill, Chapel Hill, NC USA; 2 Barefoot Ocean, LLC., Houston, Texas USA

**Keywords:** Ecology, Ecology

## Abstract

Anthropogenic climate change is intensifying natural disturbance regimes, which negatively affects some species, while benefiting others. This could alter the trait composition of ecological communities and influence resilience to disturbance. We investigated how the frequency and intensification of the regional storm regime (and likely other disturbances) is altering coral species composition and in turn resistance and recovery. We developed regional databases of coral cover and composition (3144 reef locations from 1970 to 2017) and of the path and strength of cyclonic storms in the region (including 10,058 unique storm-reef intersections). We found that total living coral cover declined steadily through 2017 (the median annual loss rate was ~ 0.25% per year). Our results also indicate that despite the observed increase in the intensity of Atlantic cyclonic storms, their effect on coral cover has decreased markedly. This could be due in part to selection for disturbance-resistant taxa in response to the intensifying disturbance regime. We found that storms accelerated the loss of threatened acroporid corals but had no measurable effect on the cover of more resilient “weedy” corals, thereby increasing their relative cover. Although resistance to disturbance has increased, recovery rates have slowed due to the dominance of small, slow-growing species. This feedback loop is locking coral communities into a low-functioning state dominated by weedy species with limited ecological or societal value.

## Introduction

The U.S. Fourth National Climate Assessment concluded that the ongoing disruption of ocean ecosystems will intensify as ocean warming, acidification, deoxygenation, and other aspects of climate change increase^1^. Due to their short-term variability, many of these environmental parameters act as acute, lethal disturbances and powerful selection agents^2–4^. The effects of climate change on natural systems are due in part to the growing frequency and intensity of acute disturbances, i.e., anthropogenic acceleration of the natural disturbance regime^5^. For example, the frequency of anomalously high ocean temperatures (AKA “marine heatwaves”) on Caribbean coral reefs has increased by fivefold and the return time has been cut in half since the mid-1980s^6–8^. This intensifying disturbance regime is causing ever more frequent mass coral bleaching events, which is driving reefs globally into a state of continual recovery, with little chance of returning to a pre-disturbance state.

The intensity (but not the frequency) of Tropical Cyclones (TCs) is predicted to increase due to the growing heat content of ocean surface waters^9–12^. Numerous studies have documented an increase in TC intensity globally (e.g., maximum wind speed and storm category) and the IPCC concluded “It is likely that the proportion of intense tropical cyclones has increased over the last four decades and that this cannot be explained entirely by natural variability”^9,13^. TCs are an important source of disturbances to coral reefs^14–17^. High winds and strong waves can fragment or uproot reef organisms or “sand-blast” the tissue off coral skeletons^18^. The ecological consequences of storms include the replacement of storm-sensitive species by weedy competitors, changes in species composition and ecosystem functioning, and a general reduction in the surface complexity of reef habitats^14,17,18^.

Despite the historical importance of TCs and the past (last ~ four decades) and forecasted near-future increase in TC intensity, recent studies have reported that the effects of TCs on coral reefs have become negligible. For example, Edmunds^19^ found that the two category 5 hurricanes that struck the reefs around St. John U.S. Virgin Islands in 2017 had no significant effects on living coral cover. Whereas, studies of the impacts of earlier category 5 hurricanes Hattie in Belize (in 1961)^15^, Allen in Jamaica (in 1980)^17^, and Hugo (1989) in the U.S. Virgin Islands^18^ describe catastrophic damage to coral communities. These findings suggest coral communities are becoming more resistant to storms and possibly other disturbances. However, the generality of and mechanisms underlying reduced hurricane effects on coral communities are unknown, particularly on contemporary, degraded Caribbean reefs.

**Figure 1:**
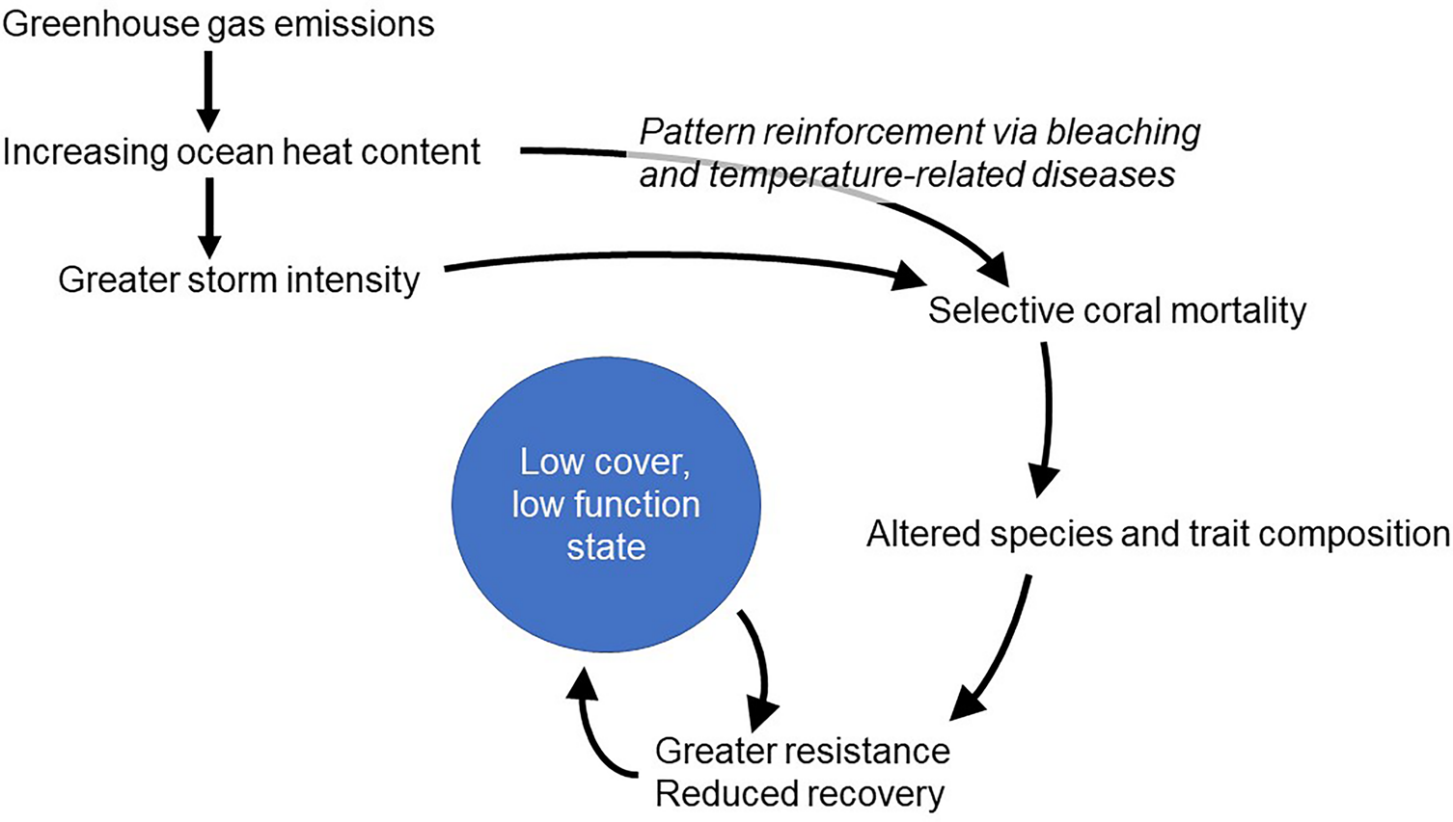
Conceptual diagram illustrating the hypothesized feedbacks between disturbance, coral species and trait composition, and resistance to and recovery from future storm events on Caribbean coral reefs in the southwest Atlantic Ocean basin.

We hypothesized that the observed increase in the resistance of coral communities to abiotic disturbances is due in part to selection for disturbance-resistant species. The intensification of the TC regime and other climate-related disturbances are negatively affecting some species, while simultaneously benefiting others^20–22^. The general pattern is selection for species with physiological, morphological, and demographic characteristics that enable them to tolerate biotic and abiotic disturbances; typically, species with “weedy” life histories^21^. The subsequent change in community trait composition could modify a wide range of ecological processes, including community responses to the disturbances driving the change. Moreover, disturbance-resistant coral species are typically slow growing and contribute little to reef accretion and habitat provision^23–25^. Such feedbacks could lock communities in a state dominated by weedy species (Fig. 1) with low ecological or societal value.

The purpose of this study was to measure the state-dependent effect of storms on Caribbean reef coral communities to better understand the causes and implications of changing coral reef resilience to storms. We quantified both resistance to (the capacity to resist change measured as the impact of a disturbance) and recovery (the rate of return to a pre-disturbance state)^26^ from TCs. Specifically, we determined whether pre-storm living coral cover and community composition affected resistance to and recovery from storm damages. Additionally, we measured functional group-specific short- and longer-term storm impacts and tested the hypothesis that changing species composition (due to the increasing intensity of the disturbance regime) is locking reefs into a resilient but low functioning state.

## Results and discussion

**Figure 2:**
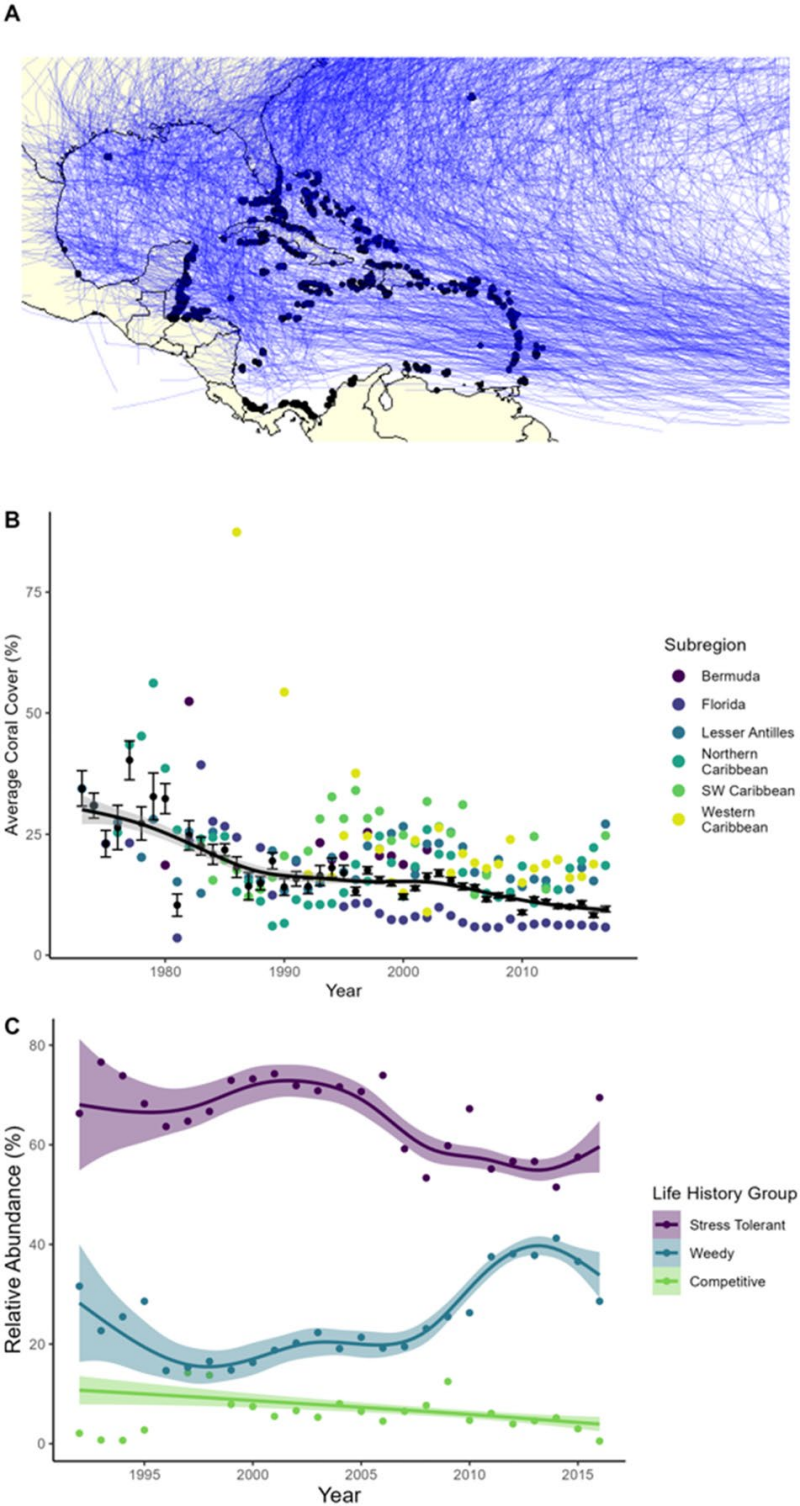
Caribbean storm and coral cover trends. (**A**) Intersection of Atlantic storm tracks from 1851 to 2017 and coral reef survey locations. Historical storm tracks are represented by blue lines and unique coral reef survey locations by black points. (**B**) Caribbean coral cover trends 1970–2017. Colored points represent subregional annual averages for scleractinian total coral cover. Black points represent basin wide annual averages in percent cover (+ / − standard error). (**C**) Trends in the relative abundance of coral life history groups during this study period (1992–2016). Points represent region-wide yearly averages.

We found that total living coral cover has steadily declined (Fig. 2B) since prior regional assessments^27–29^ which suggested that coral loss may have plateaued by the late 1990s, at a regional mean of ~ 16%. However, our results indicate that from 1997 to 2017, the median annual loss rate was ~ 0.25% per year, with a final year regional mean of 9.5% ± 0.59% (SE). The more recent regional effects of stony coral tissue loss disease^30^ including reports of severe coral loss across the Caribbean^31,32^ suggest further decline since the final year of our study. Despite substantial sub-regional variation (Fig. 2B), coral cover continues to decline, especially on reefs with relatively high coral cover. The current consensus is that this pattern is driven largely by coral bleaching, disease, TCs, and other disturbances, with local stressors including fishing and pollution playing a role at some locations^33,34^.

**Figure 3:**
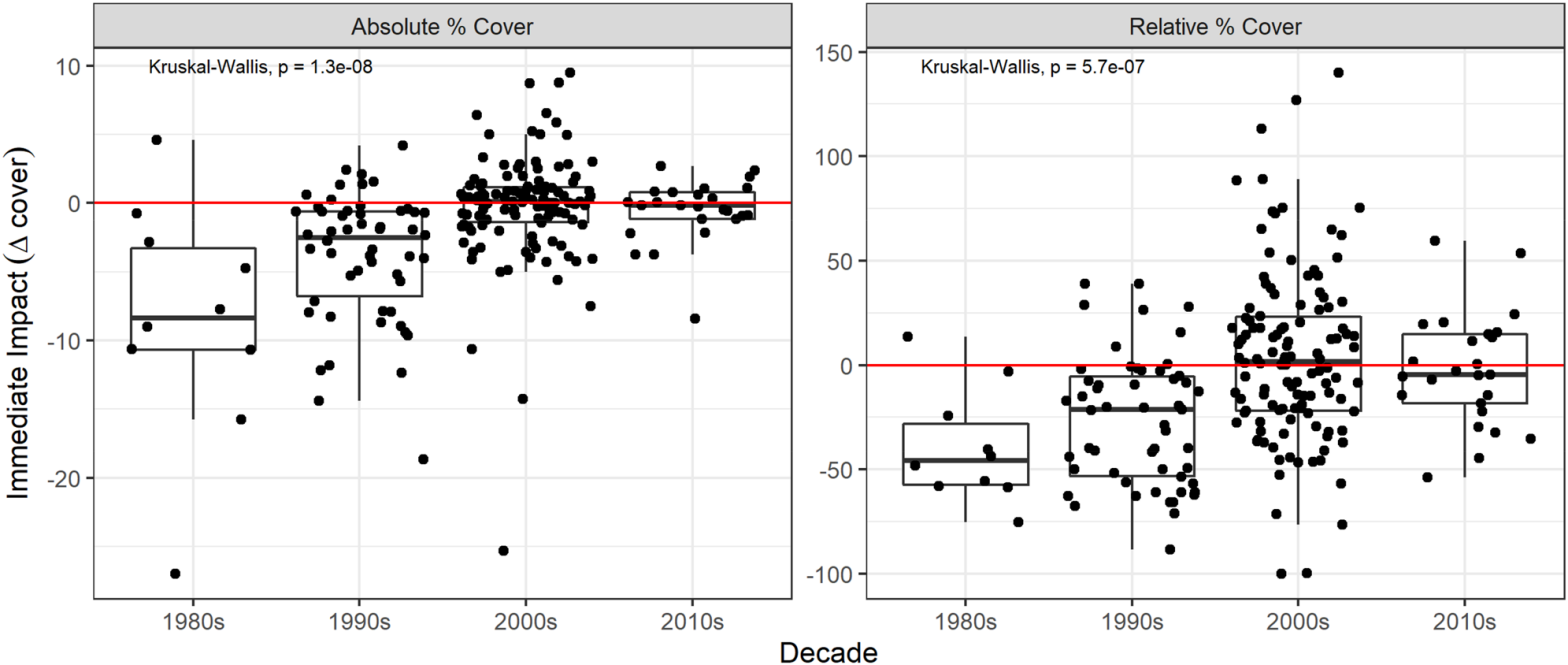
Coral resistance in the Caribbean. Decadal trends in immediate storm impacts on total coral cover. Each data point represents one storm/reef event, presented as both the change in absolute percent cover and change in percent cover relative to initial pre-storm cover.

Despite the observed increase in Atlantic storm intensity^9,13^ the immediate effect of storms on total living coral cover decreased markedly between the 1980s/1990s and the 2000s/2010s (Fig. 3). Storms now have no general immediate effect on total living coral cover (although they appear to still have subtle, longer-term effects). In contrast, our results indicate that in the 1980s, the median short-term loss was − 8.5% (for absolute total coral cover, n = 10 impacted reefs, maximum loss = 27%). Likewise, Gardner et al.^14^ found that Caribbean hurricanes between 1980 and 2001 on average caused a 17% reduction in live coral cover (n = 177 impacted reef sites). Although we have interpreted immediate post-storm changes in coral cover as storm effects, it is important to recognize that, like any natural ecosystem, coral reefs can experience multiple stressors or disturbances simultaneously. Therefore, it is possible that other factors have caused or contributed to observed post-TC changes in coral cover in some specific instances. For example, a storm strike could have coincided with bleaching event or disease outbreak^35,36^. Additionally, our method for identifying potential storm impacts could be unreliable for TCs that are either larger or smaller than normal or moving more slowly or more quickly than normal (which introduces uncertainty). Future studies should leverage methods that explicitly model storm wave damage, which would resolve these uncertainties^37,38^.

### Altered resistance and recovery

One obvious difference between the state of Caribbean reefs in the 1970s and during more recent decades is that pre-storm coral cover has been substantially reduced. Our results indicate that reefs with greater pre-impact live coral cover were less resistant (i.e., high-cover reefs lost more cover when other factors are controlled for, Supplementary Fig. S1, Supplementary Table S3). Numerous other studies have also reported this coral cover-dependent resistance to storms^14,39^, and other large-scale disturbances including disease outbreaks and bleaching events^3,39–42^. Although this phenomenon is often attributed to simply having more coral to lose, our results suggest it is being caused in part by the dominance of disturbance-sensitive species on typical high-cover reefs^33,43,44^; particularly coral species in the genus *Acropora*.

Due to their fast growth and branching or plating morphology, these species are considered competitively dominant over other sessile reef organisms, including other coral species^45,46^. Once established, acroporid corals can quickly monopolize incoming light and space on the seafloor—two critical resources for many sessile reef organisms. Such “competitively dominant” species often occupy a large majority of the substrate in ideal environments, such as those that haven’t been recently disturbed^46,47^. Only four Caribbean coral species are considered “competitive dominants”^46^, three of which are in the genus *Acropora* (*A. cervicornis, A. palmata,* and their sterile hybrid *A. prolifera)*. In U.S. waters, *Acropora* species are also listed under the Endangered Species Act as threatened. These species dominated Caribbean reefs until the early 1980s, when they were nearly extirpated by white band disease—a regional epizootic linked to ocean warming^33,44,48^. Corals in the genus *Acropora* are particularly sensitive not only to storms and other forms of physical disturbance (due to their morphology), but also to disease, predator outbreaks, and other environmental perturbations^15,46,49^; i.e., there is positive co-tolerance of sensitivities to these stressors^49,50^.

There is widespread evidence that disturbance modifies population and community responses to future disturbances through selection for resistant individuals and species^42,51,52^. The more disturbed a community is, the more resistant it becomes. The change in trait composition caused by disturbance is the mechanism underlying what is sometimes called “ecological memory”; the idea that the recent disturbance regime strongly influences current resistance and recovery^42,51,52^. On coral reefs this phenomenon is apparent across both space and time. For example, due to anthropogenic ocean warming, coral communities on Australia’s Great Barrier Reef experienced extreme temperature shocks in both 2016 and 2017, triggering widespread mass-bleaching^53^. The effect on coral cover in 2017 (even when disturbance intensity and duration were accounted for) was substantially less than in 2016^53^. Moreover, during the 2016 event, there was spatial variance in reef resistance, explained largely by pre-disturbance living coral cover (an indicator of the amount of time since the last disturbance), i.e., higher cover reefs were far less resistant^3^.

**Figure 4:**
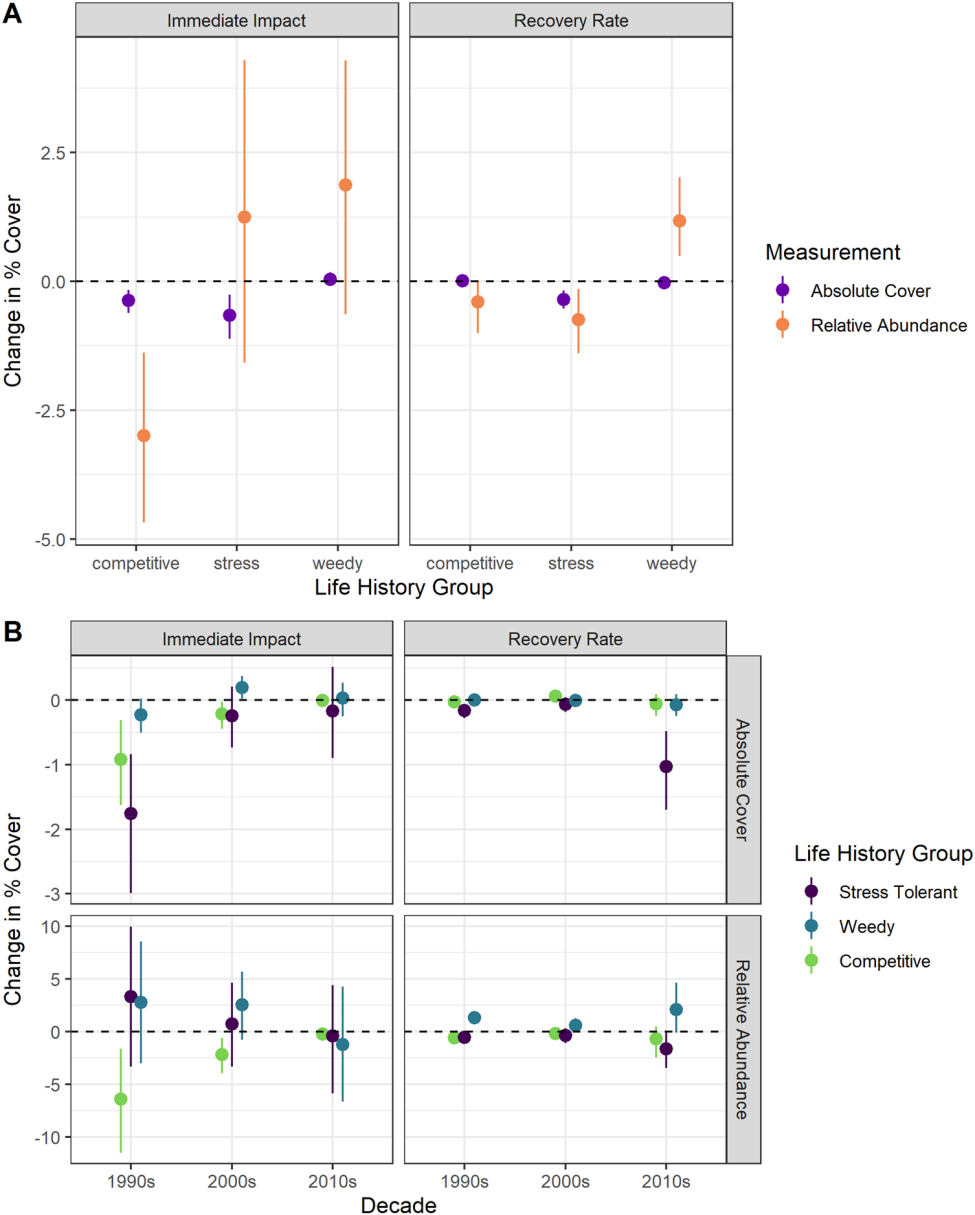
Post-storm impact and recovery rates (annual rate of change post-storm) for different coral life history groups. (**A**) Mean resistance and recovery for coral life history groups pooled across all years; (**B**) decadal means for resistance and recovery of coral life history groups. For both panels, points represent average values ± non-parametric bootstrapped confidence limits.

We found that coral functional groups displayed differential responses to TCs, based on analysis of their relative abundance (Fig. 4). In general, competitive species (as defined by Darling et al.^46^) were negatively affected by storms (mean loss per event: absolute cover = −0.4% ± 0.12%, Wilcoxon *p* = 0.009; relative cover = −3% ± 0.88%, Wilcoxon *p* = 0.003). Stress tolerant species were negatively affected by storms in terms of absolute cover (− 0.66% ± 0.22%, Wilcoxon *p* = 0.007), but due to their high abundance on many contemporary reefs, the mean change in relative abundance from pre- to post-storm was not significantly different from zero (1.24% ± 1.5%, Wilcoxon *p* = 0.92. For all groups also see Supplementary Table S4). In contrast, the cover of coral species categorized as “weedy” (e.g., *Agaricia spp.*, *Madracis* spp., and *Porites spp.*) was not affected by storms (mean change per event: absolute cover = 0.04% ± 0.07%, Wilcoxon *p* = 0.26; relative abundance = 1.87% ± 1.3%, Wilcoxon *p* = 0.09), i.e., these species were resistant to this important disturbance. Post-storm recovery rates indicate that the stress tolerant group continued to decline, likely causing the relative abundance of weedy species to increase (Fig. 4A,B).

As predicted by Côté and Darling^49^ the intensifying disturbance regime (e.g., more frequent marine heatwaves, bleaching events, storms, and disease outbreaks) on Caribbean reefs appears to have shifted reef composition into a lower cover but more resistant state (Fig. 1). This shift has increased the relative dominance (and in some subregions the absolute cover) of coral species more resistant to disturbance^24,33,54–58^. Because these weedy corals grow substantially slower (both vertically and horizontally) than competitive species^23^ overall post-disturbance recovery likely also declined. Other disturbances are likely reinforcing these changes in coral species composition, particularly mass bleaching and disease, both of which have similar species-specific effects as storms (thus reinforcing selection for weedy, disturbance-resistant species, Fig. 1). The overlap between storms, disease, and thermal stress is varied, but overall ubiquitous around the Caribbean, particularly in the Lesser Antilles/Eastern Caribbean (Fig. 2A)^7,59^. Studies that quantify multiple, interactive disturbance impacts on a regional scale could tease apart the relative contribution of disturbance events and types on these changes to coral reef communities in the Caribbean^60^.

Resistance and recovery need not be positively related across sites and often aren’t. For example, Patrick et al.^61^ found that there was a strong and general (across multiple terrestrial and aquatic habitat types) negative association between system resistance and recovery in response to TCs. A negative covariance between resistance and recovery can be due to evolutionary trade-offs between the characteristics that influence a species’ responses to disturbance^61,62^. Our results suggest that there is a trade-off between resistance and recovery for Caribbean coral communities. This characteristic of modern coral reefs (and possibly other marine systems) underlies the protection paradox; when by promoting high coral cover and fast-growing resilient species (e.g., via coral gardening of *Acropora spp.)* managers inadvertently reduce community resistance to the next inevitable disturbance^4,49^.

By increasing resistance and slowing recovery, an intensified storm regime appears to be contributing to a feedback loop, locking reef communities into a low functioning state (Fig. 1). The traits of coral species determine their functional roles and the overall ecosystem functions of the coral community^63,64^. The observed compositional shift toward dominance by weedy coral species also degrades other important reef characteristics and functions including vertical reef accretion, surface complexity, and habitat provision^54,65^. In the Caribbean competitive coral species and some stress tolerant species in the genus *Orbicella* disproportionately contribute to increased structural complexity and calcification^23^. In contrast, weedy species generally have only minimal influence on these functions due to their smaller colony sizes, flatter morphologies, and slower rates of calcification and vertical reef accretion^23,24^. The loss of structural complexity is especially important due to the established links between complexity and multiple ecosystem services including the facilitation of community inhabitants such as invertebrates and fishes and subsequently of fisheries productivity^24,65^.

### Long-term storm effects

**Figure 5:**
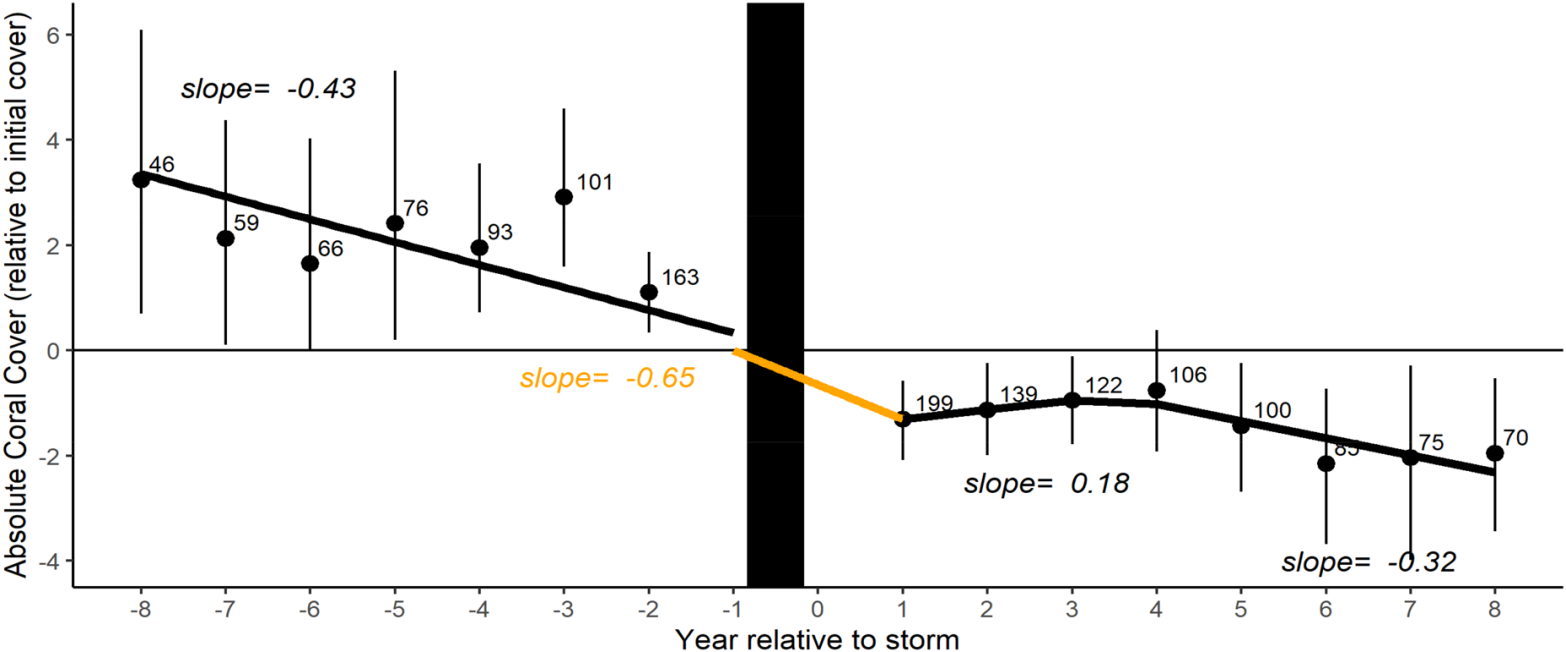
Average yearly change in coral cover, relative to year of impact. The vertical black bar represents a storm event on a reef. Black dots are Caribbean-wide average change in coral cover for any year pre/post storm (± bootstrapped confidence intervals), relative to initial cover (percent cover at one year prior to storm). Numbers represent sample sizes for averages. Lines represent the slope of change in coral cover pre- and post-storm, with the orange line representing immediate impact.

Although the immediate effects of storms on coral communities have declined, the longer-term impacts appear to linger for years (Fig. 5). Like Gardner et al.^14^, we found little evidence of post-storm recovery. Instead, we found a brief period of stasis after the immediate impact of a storm, and then resumption of pre-storm decline (2005). This pattern could be due to delayed disease or coral predator outbreaks triggered by the storm^66,67^. For example, physical damage from tropical storms, such as coral fragmentation and increased sedimentation, may provide more opportunity for contact between pathogens and live coral tissue^35^.

**Figure 6:**
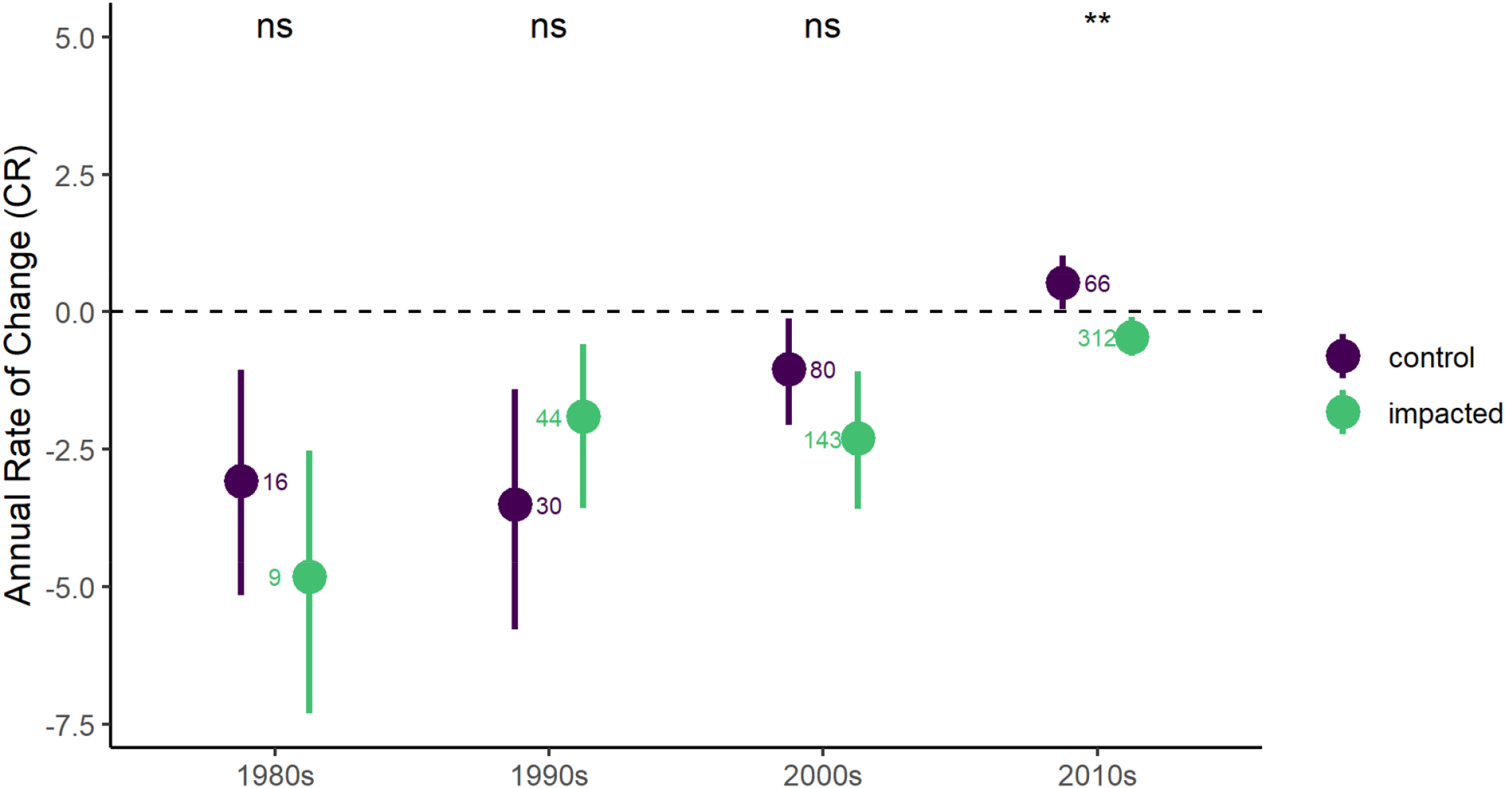
Annual rate of change in absolute coral cover by decade at the study sites. Points represent Caribbean-wide averages (± bootstrapped confidence intervals, values adjacent to each point represent sample sizes − number of sites − for each period and category). “Control” sites were not exposed to potential impact from a storm during the study period (see “Methods” section “Control reefs” for additional details).

When comparing the average rate of change in coral cover between storm-impacted and unimpacted sites, we found that storm-impacted sites still have a negative recovery rate (i.e., continued decline post-storm) in the 2010s while unimpacted sites have a slightly positive rate of change, indicating recovery from other disturbances, likely including coral bleaching and disease (Fig. 6). Coral reefs experience a simultaneous myriad of disturbances and stressors, including storms, thermal stress (leading to coral bleaching), disease, and localized impacts from pollution and fishing. Therefore, it is possible that concurrent or subsequent disturbances or stressors interact with storm impacts to further exacerbate coral cover decline. For example, synergistic interactions between hurricanes and coral disease, in which storm-damaged sites have a higher prevalence of coral disease compared to unimpacted sites, has been documented on Caribbean reefs^36,68,69^. Thus, the effect of storms on cover, composition, and functioning often continue for a significant period after the event.

## Conclusion

The contribution of storm impacts to short-term coral decline in the Caribbean has decreased over the past few decades; however, localized immediate impacts from storms can still be substantial on reefs with high coral cover or longer time intervals between storm events. Storms, combined with thermal stress and disease, appear to be driving a shift towards coral assemblages dominated by resistant but low-functioning weedy species (Fig. 2C). Increasing trends in storm intensity have important regional implications, not only for natural marine and terrestrial ecosystems and wildlife, but also for people. More, stronger storms in the Caribbean will result in increased damage to infrastructure and loss of ecosystem services that humans rely on.

## Methods

### Summary

We compiled a coral reef-storm dataset that catalogues the historical record of storm crossings on coral reefs in the greater Caribbean and Gulf of Mexico (Fig. 2A). This database consists of 11,490 quantitative benthic surveys from 3144 unique reef locations performed between 1970 and 2017. Survey data was compiled from various coral reef monitoring programs and the literature (Supplementary Table S1) and each survey reported the absolute living coral cover as a percentage of the benthic composition. A subset of the data sources also reported the percent cover of individual coral species, which we aggregated to determine the percent cover of three ecological functional groups (weedy, competitive, and stress tolerant, see Supplementary Table S2) across 672 unique reef locations. We then combined these coral community data with the tracks of Atlantic storms (tropical storm strength or stronger) sourced from the National Oceanic and Atmospheric Administration (NOAA)’s HURDAT2 dataset^71^. For the 47 years for which we had coral survey data there were 547 storms, 152 of which crossed at least one surveyed reef, resulting in 10,058 unique storm-reef intersections. When coral reef survey data was available for pre- and post-storm years at a site (1 year prior and 1 year post), we quantified the effect of storms on living coral cover and community structure, and their dependency on pre-impact reef state.

### Coral survey data acquisition

Coral reef benthic survey data was obtained from primary source databases, peer-reviewed literature, and grey literature. Databases included widely-used and publicly available coral reef monitoring programs, such as Reef Check, Reef Life Survey, and the Atlantic and Gulf Rapid Reef Assessment (AGRRA). Primary databases with download date are listed in Supplementary Table S1. This study combined a previous version of a Caribbean coral cover database used for analysis in Selig and Bruno^72^ and Schutte et al.^27^) with more recent coral survey data from longitudinal studies and monitoring datasets. We relied heavily on data from monitoring programs because they provide large amounts of repeated measurements over long time frames and cover broad spatial scales, both of which are essential for making conclusions regarding regional trends and possibly help mitigate the effects of publication bias. Most surveys were not conducted on permanent transects, and GPS coordinates were used to verify the location on the reef for resurveying over time. All monitoring programs collect data on characteristics of the habitat surveyed (e.g., reef zone, such as bank reef or patch reef), which aids in verifying that the same reef area is resurveyed.

Absolute living scleractinian coral cover was measured using quantitative techniques including line transect intercept (in-situ counts along a transect line) and point count (randomized points taken from video transects or photo quadrats). Despite differences in survey methodology and possibly precision and accuracy, the metric of percent cover of the benthos is recognized as a fairly coarse measurement, resulting in negligible differences that are not statistically affected by the method of collection^73^. The data collection methods for obtaining coral species data were similar (AGRRA or modified-AGRRA protocols) and conducted by trained scientists.

Studies were included if they reported a reef site location, survey date (or year), and a measure of absolute percent cover for scleractinian corals. When latitude and longitude coordinates of survey locations were not provided in the reference, we used site location descriptions and maps from the text to identify approximate coordinates using Google Earth, when possible. In addition to manual data entry from primary literature, three tools were used to extract data from pdf resources: the tabulizer package in R^74^ was used to extract raw percent cover data from tables, and ImageJ (from previous Bruno lab database only, see Schutte et al., 2010) and/or Web Digitizer^75^ was used to extract raw percent cover data from figures. If more than one survey was conducted at the same reef site on any given day, percent coral cover was averaged to produce one value per day/location combination. Coral reef survey locations were considered unique based on the latitude and longitude coordinates provided from the dataset or study. The resulting database includes survey data from 3144 unique reef locations throughout the Caribbean with 11,490 measurements of coral cover between 1971 and 2017. Approximately 23% of the data came from peer-reviewed literature sources and 77% from coral reef monitoring programs.

### Coral cover by life history group

Absolute percent cover of distinct coral species was obtained from three sources, mostly focused in Florida and the US Virgin Islands (Supplementary Table S1, sources with **). Coral species were assigned a life history group (LHG) of either competitive, stress tolerant, or weedy based on classifications made in Darling et al.^46^. These assignments are based on qualities related to species specific growth and reproduction (Supplementary Table S2). Coral species not yet assigned to a LHG were labeled as “unclassified”. The relative cover for each life history group was calculated by site and year using the calculation:

$$Relative\, \%\, Cover\, of\, LHG= \frac{Absolute\, \%\, Cover\, of\, LHG}{Total\, Coral\, \%\, Cover}\, x\, 100$$

### Building a hurricane and coral reef intersection database

Historical storm track data was downloaded directly from the National Oceanic and Atmospheric Administration (NOAA) Atlantic Hurricane Database (HURDAT2) using the HURDAT package in R^76^. These historical records contain storm track location (latitudinal and longitudinal coordinates), wind speed (knots), low pressure (millibar), status (landfall, hurricane classification), date and time, with variables recorded every 6-h. Historical track information from the earliest year (1851) to present was used to analyze overall storm patterns in the Atlantic basin.

Functional programming in R was used to catalog which hurricanes cross which reef sites in the coral reef survey dataset. Code for these procedures was adapted from Elsner and Jagger^77^. For each reef, we searched for all historical storms occurring within a 100km radius of the reef site coordinates. Storms of any strength were retained within a 35km radius of the reef coordinates, storms of category 3–5 on the Saffir-Simpson scale were further retained between 35 and 60km, and only category 4 and 5 storms retained between 65 and 100km. These buffers are based on previously published hurricane path impacts to coral reefs^78,79^. It is possible that our buffers could have excluded storms that affected far away reefs (e.g., a large and/or slow-moving storm) or could have included storms that did not generate damaging waves for the full 100km (e.g. a small and/or fast moving storm)^38,80,81^. Methods to more accurately predict storm wave damage can be found in Puotinen et al.^37,38^. Each observation in the database is a unique reef-storm intersection. Therefore, reef locations appear multiple times in the database, if more than one storm has hit the reef since 1851, and individual storms appear multiple times if they struck multiple coral reef locations along their path.

It is also important to note that numerous factors in addition to wind speed and storm size influence the realized effects of storms on coral communities, e.g., the local bathymetry, depth, speed at which storm moves along its track, and the angle of orientation of the shorelines, etc.^38,80,81^. Additionally, spatial variability in coral community response to hurricanes exists both between and within storm events. Hurricanes can result in substantial coral mortality, including complete decimation of coral populations in some cases, and changes to the physical reef structure^45,82–85^. However, there are also documented instances of minimal structural damage or reduction in coral cover from hurricanes^19, 86^. Even within a single storm event at a single location the coral community response may vary across a reef landscape, including at the scale of only a few hundred meters^87^. Other studies also demonstrate a wide range of differential impacts (e.g., difference in coral cover loss 30–70%) between nearby sites hit by the same storm^17,44, 83,86,88^. This spatial variability in coral response can be a result of the individual storm characteristics (intensity, frequency, duration), site-specific disturbance history, habitat heterogeneity (reef depth, profile/slope, exposure), environmental gradients along reef sites, or differences in the initial conditions (coral cover, species abundance and diversity) at a reef site^14,82,83,86,87,89^. All of these factors contribute to patchiness in storm impacts on coral reefs. We attempted to quantify many of these as predictors in our statistical models (see section on linear mixed effects models below and Supplement Tables S3 and S4), but ultimately recognize that this study does not account for all potential sources of local-scale variability and patchiness to storm impacts.

Historically (1851–2017), approximately 32% of named storms in the southwestern Atlantic Ocean basin have hit a coral reef location (1604 named storms, 521 hit a reef). Between 1970 and 2017, the time period of coral survey sampling, there were 547 storms total, 28% of which crossed over at least one coral reef site, for a total of 10,058 unique site-storm intersections. Out of 3144 unique coral reef survey sites, 2754 sites experienced at least one tropical storm since the beginning of storm records in 1851 (87.6% of reefs impacted, 12.4% of reefs unimpacted). Sites that were not impacted were located in the SW Caribbean, along the coast of Panama, Colombia, and Costa Rica.

For each unique reef site, we calculated several measurements pertaining to the disturbance regime of tropical storms, including the total number of storms to ever hit that reef, historical return time (average number of years between storm events), storm dispersion patterns, and the average historical maximum intensity of all storms, weighted by their distance to the reef. All of these variables were calculated from coral-storm intersections that occurred between 1851 and 2017. The dispersion statistic is used to assess the temporal clustering of hurricanes and has demonstrated ecological impacts on coral reef ecosystems^90^. Using previously described methods, we tabulated a count vector (Y) of storm events per reef for each year between 1851 and 2017. The dispersion statistics (Ψ) is calculated as:

$$\uppsi \left(Y\right)=\frac{variance (Y)}{mean (Y)}-1$$

Storm dispersion patterns were characterized as follows: Stochastic (random): Ψ (Y) = 0 (i.e. variance = mean); Clustered (over-dispersed): Ψ (Y) > 0 (i.e. variance > mean); Regular (under-dispersed): Ψ (Y) < 0 (i.e. variance < mean).

### Control reefs

A subset of the larger coral cover database was identified to serve as a “control” dataset. This included coral cover data from sites that were either (1) never hit by a storm (as determined with our buffer methods); (2) had a substantial amount of time (> 10 years) between storm events. As previously mentioned, uncertainties exist in the buffer methods, particularly for capturing exposure to large/slow-moving or small/fast-moving storms, and therefore it’s possible some ‘control’ sites were actually impacted.

For sites that had been hit by a storm, coral cover data was only retained for a period of 10 years after a previous storm until the next storm hit. This is to ensure that we were not including potential storm recovery trajectories as part of a control condition. For each reef site, we calculated the annual rate of change in coral cover (CR) to use as a comparison against the rate of change in coral cover at storm-impacted sites^7^. The CR value was also calculated for each Caribbean subregion in order to account for anticipated spatial variation in coral cover and potential local conditions contributing to coral decline.

### Quantifying resilience

#### Resistance

Coral resistance to tropical storm damage was measured as the change in coral cover from initial conditions (one year prior to a storm) and one-year post-storm and is presented as both the absolute change, and change relative to the initial cover. Relative resistance was calculated as:

$$Relative\, resistance =\frac{(\%\, cover\, post \,storm - \%\, cover\, pre\text{-}storm)}{\%\,cover\, pre\text{-}storm}\, \times \, 100$$

((% cover post storm − % cover pre-storm)/%cover pre-storm) × 100.

Paired Wilcoxon tests were used to quantify differences in cover before and after each individual storm event at each reef (i.e. each site-storm combination is one observation for this test). A Kruskal–Wallis test was used to test the hypothesis that coral resistance is greater (meaning less coral loss from storms) in more recent decades.

#### Recovery

Temporal patterns in coral recovery were quantified in two ways: as (1) the relative change in coral cover at any year pre- or post-storm, relative to coral cover in the year preceding a storm, here referred to as the initial conditions; and (2) as the annual rate of change in absolute coral cover (CR), post-storm^91^.

1. Relative recovery = % cover at year relative to storm − % cover before impact.
2. Annual rate of change in coral cover (CR) = (pca-pcb)/d.

Quantifying the relative change in coral cover for years both before and after a storm event allows us to compare the impact of storms on pre-disturbance trends. First, we used regression models to evaluate the trend in relative recovery for the time periods pre- and post-storm. Upon visual review of linear regression (using ordinary least squares models), it became apparent that one linear relationship did not persist throughout the time period of recovery, but rather multiple piecewise relationships might exist. We used the segmented package in R to estimate the appropriate breakpoints for the regressions^92^. We then compared the slopes in the piecewise regressions for several time periods pre- and post-storm to describe patterns of recovery. Next, we quantified the annual rate of change in coral cover (CR) after a storm event. CR is measured over each individual storm event time series, in which pca is the percent cover at the end of the time series, pcb is the coral cover immediately after a storm (post one-year), and d is the duration of the time series, calculated as the number of years between pcb and pca. If two or more storms occurred in the same year/site, the CR time series was kept for the stronger storm and/or later storm. Resistance and recovery were quantified for both absolute coral percent cover (all species) and the relative abundances of coral life history groups. In text, resistance and recovery results are reported as mean ± standard error.

Linear mixed models were used to quantify the effects of a variety of disturbance characteristics on coral resistance and recovery. Predictors included a mix of event specific characteristics and disturbance regime characteristics (Supplementary Table S3). All predictors were treated as fixed effects, except for reef location, which was treated as a random effect to account for variation amongst individual reef sites. Prior to modeling, raw data were analyzed for normality, heteroscedasticity, outliers, and collinearity via pairs plots and variance inflation factors (VIF). Predictors with a VIF > 2 were removed from the model. Historical return time and the historical number of storms were collinear and had high VIF and for each model, whichever variable had the higher VIF was removed. In the resistance models for coral life history groups, storm distance was also removed due to high collinearity with wind speed and high VIF. Response variables had a non-normal distribution and included both zeros and negative values, so a cube-root transformed was performed prior to modeling. All continuous fixed effects were scaled prior to modeling. Model residuals were also evaluated to meet assumptions of normality and homoscedasticity. Models were run using the lme4 package^93^ in R. All analyses were conducted in R version 3.6.1.

### Preprint

A previous version of this manuscript was published as a preprint^94^.

### Supplementary Information

Supplementary Information.

## Data Availability

The datasets analyzed in the current study are available at: https://github.com/Lmudge13/Caribbean_Coral_Reef_Hurriane_Impacts. The compiled raw coral cover data set is available from the corresponding author on reasonable request; however, this dataset is not re-printed or publicly available due to license and user agreements from individual databases. All databases included in the coral cover dataset can be accessed using the references provided in Supplementary Table S1.
