# Supplementary material for: Disturbance intensification is altering the trait composition of Caribbean reefs, locking them into a low functioning state
Source: Sci Rep. 2023 Aug 28;13:14022. doi: 10.1038/s41598-023-40672-x (PMC10462730; doi:10.1038/s41598-023-40672-x)
Supplement: Supplementary file 1 — Supplementary Information. [file 41598_2023_40672_MOESM1_ESM.pdf]

Supplementary Information for

Disturbance intensification is altering the trait composition of Caribbean reefs, locking them into a low functioning state.

Laura Mudge[1,2]*†, John F. Bruno[1]

Laura Mudge
Email: lmudge13@gmail.com

This PDF file includes:

Figures S1
Tables S1 to S4
SI References

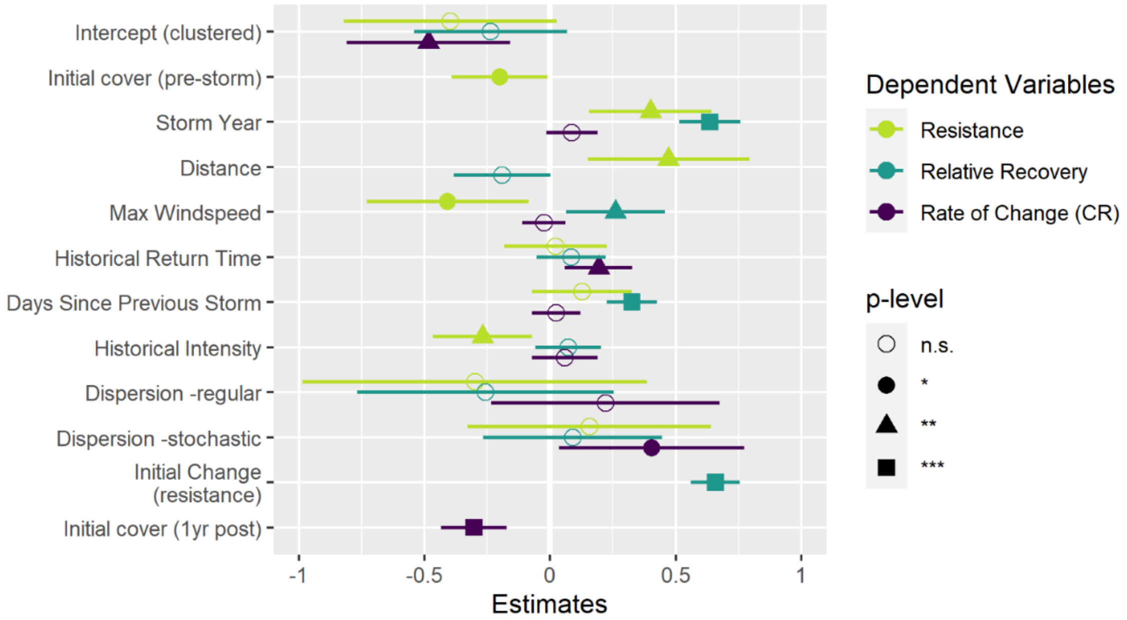

Supplementary Figure S1. Effect of storm parameters of coral resistance and recovery. Model estimates are from linear mixed effects models in which all predictors were treated as fixed effects and site as a random effect. Dependent variables were cube root transformed and all fixed effects scaled prior to modeling. Signif. codes: 0 '***' 0.001 '**' 0.01 '*' 0.05 '.' 0.1 ' ' 1

Supplementary Table S1. Data sources for coral cover database. (**) Indicates sources that also had coral percent cover for individual species.

Database or monitoring program	Date of data access or download	Reef locations surveyed	Years of data coverage
Bruno Lab database[1]: includes earlier reef check, AGRRA, and literature sources	March 2017	Region wide	1971-2006
Atlantic and Gulf Rapid Reef Assessment[2] **	March 2018	Region wide	1998-2016
Florida Fish and Wildlife Conservation Commission: Coral Reef Evaluation and Monitoring Project (CREMP)[3] **	January 2019	Florida	1996-2017 (coral cover) 1992-2015 (species cover)
Reef Check[4]	March 2018	Region wide	1997-2017
Reef Life Survey[5]	August 2018	Region wide	2010-2012
NSF Coral Time Series, Virgin Islands[6]	June 2019	USVI	1987-2015
CSUN and NPS USVI time series[7] **	August 2018	Guest et al 2018	2003-2015
US Virgin Islands Territorial Coral Reef Monitoring Program (TCRMP)[8] **	September 2019	USVI	2001-2010 (coral cover) 2001-2016 (species cover)
CARICOMP[9]	July 2019	Region wide	1993-2003
Stokes et al., 2010[10]	July 2019	Bonaire	1982-2008
Steneck et al., 2019[11]	July 2019	Bonaire	2004-2017
Toth et al., 2014[12]	July 2019	Florida	1998-2011

Supplementary Table S2. Classification of coral species by life history group.

**Competitive Species**	
Acropora cervicornis	*Acropora palmata*
Acropora prolifera	*Acropora sp.*
**Stress Tolerant**	
Colpophyllia natans	*Dichocoenia stellaris*
Dichocoenia stokesii	*Diploria labyrinthiformis*
Diploria sp.	*Eusmilia fastigiata*
Favia fragum	*Favia sp.*
Meandrina meandrites	*Montastraea cavernosa*
Montastraea sp.	*Orbicella annularis*
Orbicella faveolata	*Orbicella franksi*
Orbicella sp.	*Pseudodiploria clivosa*
Pseudodiploria sp.	*Pseudodiploria strigosa*
Siderastrea siderea	*Stephanocoenia intersepta*
**Weedy species**	
Agaricia agaricites	*Agaricia fragilis*
Agaricia grahamae	*Agaricia humilis*
Agaricia lamarcki	*Agaricia sp.*
Agaricia tenuifolia	*Agaricia undata*
Isophyllia sinuosa	*Madracis auretenra (mirabilis)*
Madracis decactis	*Madracis formosa*
Madracis pharensis	*Madracis sp.*
Manicina areolata	*Mycetophyllia ferox*
Porites astreoides	*Porites cf. branneri*
Porites divaricata	*Porites furcata*
Porites porites	*Porites sp.*
Siderastrea radians	

Supplementary Table S3. Effect of storm parameters on coral resistance and recovery. Estimated regression parameters, standard errors, confidence intervals, t-values, and p-values from linear mixed effects models on percent coral cover. For all models, reef location was treated as a random effect.

Response Variable & Fixed effects	Estimate	Standard Error	Confidence Intervals	t-value	p-value	Direction of slope
**Immediate Impact**						
(Intercept)	-0.404	0.216	-0.82 – 0.03	-1.832	0.067	
Initial Cover	-0.219	0.097	-0.39- -0.01	-2.056	**0.040 ***	neg
Storm Year	0.397	0.124	0.16- 0.64	3.206	**0.001 *****	pos
Distance	0.489	0.164	0.15- 0.79	2.869	**0.004 *****	pos
Max Wind Speed	-0.412	0.163	-0.73- -0.09	-2.483	**0.013 ***	neg
Historical Return Time	0.025	0.104	-0.18- 0.23	0.207	0.836	
Dispersion (regular)	-0.312	0.350	-0.98- 0.39	-0.853	0.394	
Dispersion (stochastic)	0.134	0.247	-0.33- 0.64	0.635	0.526	
Days Since Previous Storm	0.128	0.100	-0.07- 0.32	1.258	0.208	
Historical Storm Intensity	-0.276	0.100	-0.47- -0.07	-2.661	**0.008 *****	neg
Marginal R^2= 0.244; Conditional R^2 = 0.279; Random effects variance: σ^2 = 1.36; *n= 209*						
**Relative recovery (up to 8 years post-storm)**						
(Intercept)	-0.23	0.15	-0.52 - 0.06	-1.56	0.12	
Storm Year	0.64	0.06	0.52 - 0.76	10.28	**< 0.001 *******	pos
Distance	-0.17	0.10	-0.36 - 0.02	-1.77	0.08	
Max Wind Speed	0.24	0.10	0.05 – 0.43	2.44	**0.015 ***	pos
Days Since Previous Storm	0.33	0.05	0.23 - 0.42	6.53	**< 0.001 *******	pos
Historical Return Time	0.05	0.07	-0.08 - 0.18	0.70	0.49	
Historical Storm Intensity	0.04	0.06	-0.08 - 0.17	0.69	0.49	
Initial Change (resistance)	0.67	0.05	0.58 - 0.77	13.69	**< 0.001 *******	pos
Dispersion (regular)	-0.32	0.25	-0.81 - 0.18	-1.26	0.21	
Dispersion (stochastic)	0.04	0.17	-0.30 – 0.38	0.23	0.82	
Marginal R^2= 0.482; Conditional R^2 = 0.651; Random effects variance: σ^2 = 0.73; *n= 866*						
**Annual rate of change (CR)**						
(Intercept)	-0.483	0.166	-0.81 – -0.16	-2.908	**0.004 *****	neg
Storm Year	0.088	0.052	-0.014 - 0.189	1.698	0.089	
Max Wind Speed	-0.024	0.044	-0.111 - 0.062	-0.552	0.581	
Days Since Previous Storm	0.026	0.049	-0.070 - 0.122	0.522	0.602	
Historical Return Time	0.193	0.069	0.058 - 0.328	2.810	**0.005 *****	pos
Historical Storm Intensity	0.059	0.066	-0.071 - 0.189	0.889	0.374	
Initial Cover	-0.303	0.067	-0.434 - -0.172	-4.526	**< 0.001 *******	neg
Dispersion (regular)	0.221	0.232	-0.234 - 0.676	0.951	0.341	
Dispersion(stochastic)	0.405	0.188	0.037 - 0.774	2.154	**0.031***	pos
Marginal R^2 = 0.164; Conditional R^2= 0.751; Random effects variance: σ^2 = 0.25; n= 282						

Signif. codes: 0 '*' 0.001 '**' 0.01 '*' 0.05 '.' 0.1 ' ' 1**

Supplementary Table S4. Effect of storm parameters on resistance and recovery to coral life history groups. Estimated regression parameters, standard errors, confidence intervals, t-values, and p-values from the linear mixed-effects models on resistance and recovery of coral life history groups.

Response Variable & Fixed effects	Estimate	Standard Error	Confidence Intervals	t-value	p-value	Direction of slope
**Resistance**						
(Intercept)	0.252	0.565	-0.86-1.36	0.445	0.656	
Initial Cover	-1.123	0.101	-1.321- -0.925	-11.118	**< 0.001 *****	neg
Storm Year	0.023	0.077	-0.128 - 0.174	0.300	0.764	
Max Wind Speed	-0.072	0.080	-0.229 - 0.084	-0.904	0.366	
Days Since Previous Storm	-0.053	0.082	-0.213 - 0.107	-0.644	0.520	
Historical Return Time	-0.020	0.080	-0.177 - 0.137	-0.249	0.804	
Historical Storm Intensity	-0.002	0.078	-0.155 - 0.151	-0.024	0.981	
Dispersion (regular)	-0.353	0.291	-0.923 - 0.218	-1.212	0.225	
Dispersion (stochastic)	-0.269	0.215	-0.690 - 0.153	-1.250	0.211	
Marginal R^2 = 0.268, Conditional R^2 = 0.506, Random effects variance: σ^2 = 2.35, n =568						
**Recovery (Annual Rate of Change, CR): Stress-tolerant group**						
(Intercept)	-0.194	0.329	-0.84-0.45	-0.590	0.555	
Storm Year	6.89E-04	0.085	-0.166 - 0.167	0.008	0.994	
Max Wind Speed	0.0014	0.089	-0.173 - 0.176	0.016	0.988	
Days Since Previous Storm	0.0524	0.092	-0.127 - 0.232	0.571	0.568	
Historical Return Time	-0.2906	0.144	-0.572 - -0.009	-2.024	**0.043 ***	neg
Historical Storm Intensity	0.0326	0.116	-0.196 - 0.261	0.280	0.779	
Initial post-disturbance cover	-0.7054	0.107	-0.915 - -0.496	-6.596	< **0.001 *****	neg
Dispersion (regular)	-0.2412	0.418	-1.060 - 0.578	-0.577	0.564	
Dispersion (stochastic)	-0.1028	0.381	-0.850 - 0.644	-0.270	0.787	
Marginal R^2 = 0.237, Conditional R^2 = 0.660, Random effects variance: σ^2 = 0.71, n =176						
**Recovery (Annual Rate of Change, CR): Competitive group**						
(Intercept)	-0.238	0.190	-0.61-0.13	-1.251	0.211	
Storm Year	0.064	0.048	-0.030 - 0.158	1.328	0.184	
Max Wind Speed	0.016	0.050	-0.082 - 0.114	0.321	0.748	
Days Since Previous Storm	0.033	0.052	-0.069 - 0.134	0.634	0.526	
Historical Return Time	0.233	0.084	0.068 - 0.399	2.766	**0.006 ****	pos
Historical Storm Intensity	0.014	0.067	-0.117 - 0.146	0.215	0.830	
Initial post-disturbance cover	-0.428	0.062	-0.550 - -0.306	-6.878	< **0.001 *****	neg
Dispersion (regular)	-0.040	0.241	-0.512 - 0.432	-0.165	0.869	
Dispersion (stochastic)	0.274	0.221	-0.160 - 0.707	1.238	0.216	

Marginal R^2 = 0.260, Conditional R^2 = 0.689, Random effects variance: σ^2 = 0.22, n =175						
**Recovery (Annual Rate of Change, CR): Weedy group**						
(Intercept)	0.533	0.299	-0.05-1.12	1.780	0.075	
Storm Year	-0.143	0.079	-0.298 - 0.011	-1.821	0.069	
Max Wind Speed	0.006	0.078	-0.146 - 0.158	0.074	0.941	
Days Since Previous Storm	-0.104	0.080	-0.260 - 0.052	-1.304	0.192	
Historical Return Time	0.066	0.129	-0.187 - 0.319	0.509	0.611	
Historical Storm Intensity	0.010	0.105	-0.195 - 0.215	0.097	0.922	
Initial post-disturbance cover	-0.528	0.097	-0.718 - -0.339	-5.461	**< 0.001 *****	neg
Dispersion (regular)	0.290	0.378	-0.451 - 1.030	0.766	0.444	
Dispersion (stochastic)	-0.179	0.349	-0.863 - 0.505	-0.513	0.608	
Marginal R^2 = 0.230, Conditional R^2 = 0.679, Random effects variance: σ^2 = 0.53, n =175						

Signif. codes: 0 '*' 0.001 '**' 0.01 '*' 0.05 '.' 0.1 ' ' 1**

Supplementary References

1. Schutte, V. G. W., Selig, E. R., and Bruno, J. F. (2010). Regional spatio-temporal trends in Caribbean coral reef benthic communities. *Mar. Ecol. Prog. Ser.* 402, 115–122. doi:10.3354/meps08438.
2. AGRRA. 2019. Atlantic and Gulf Rapid Reef Assessment (AGRRA): An online database of AGRRA coral reef survey data. Available: http://agrra.org. (Accessed: 2018-3-13)
3. Florida Fish and Wildlife Conservation Commission- Fish and Wildlife Research Institute and CREMP Team and Collaborators. Coral Reef Evaluation and Monitoring Project. Available: https://geodata.myfwc.com/datasets/60c6e6651a774b0aa40adbe8e5c68ae1_13/explore (Accessed: 2019-1-16)
4. Reef Check Foundation. Reef Check Global Reef Dataset. Available: data.reefcheck.org. (Accessed: 2018-3-21)
5. Edgar, GJ and Stuart-Smith, RD 2018., Reef Life Survey (RLS): Habitat Quadrats. Institute for Marine and Antarctic Studies (IMAS). Available at https://catalogue-rls.imas.utas.edu.au/geonetwork/srv/en/metadata.show?uuid=6e9c4980-1005-11dd-b28e-00188b4c0af8. (Accessed 2018-8-10)
6. California State University Northridge and P. Edmunds. 2019. Virgin Islands National Park: Coral Reef: Population Dynamics: Scleractinian corals ver 1. Environmental Data Initiative. https://doi.org/10.6073/pasta/8cd311eae47675f714717fe78c737634 (Accessed 2019-06-11).
7. Guest, J.R., Edmunds, P.J., Gates, R.D., Kuffner, I.B., Brown, E.K., Rodgers, K.S., Jokiel, P.L., Ruzicka, R.R., Colella, M.A., Miller, J., Atkinson, A., Feeley, M.W., Rogers, C.S., 2018, Time-series coral-cover data from Hawaii, Florida, Mo'orea, and the Virgin Islands: U.S. Geological Survey data release, https://doi.org/10.5066/F78W3C7W (Accessed: 2018-8-10)
8. TCRMP Benthic Cover and Coral Health datasets. Available at: https://sites.google.com/site/usvitcrmp/home (Accessed: 2019-9-25)
9. Linton, D., and Fisher, T. (2004). CARICOMP: Caribbean coastal marine productitity program: 1993-2003. , eds. D. Linton and T. Fisher Caribbean Coastal Marine Productivity (CARICOMP) Program.
10. Stokes, M. D., Leichter, J. J., and Genovese, S. J. (2010). Long-Term Declines in Coral Cover at Bonaire, Netherlands Antilles. *Atoll Res. Bull.* 582, 1–21.
11. Steneck, R. S., Arnold, S. N., Boenish, R., de León, R., Mumby, P. J., Rasher, D. B., et al. (2019). Managing recovery resilience in coral reefs against climate-induced bleaching and hurricanes: A 15 year case study from Bonaire, Dutch Caribbean. *Front. Mar. Sci.* 6, 1–12. doi:10.3389/fmars.2019.00265.
12. Toth, L. T., van Woesik, R., Murdoch, T. J. T., Smith, S. R., Ogden, J. C., Precht, W. F., et al. (2014). Do no-take reserves benefit Florida's corals? 14 years of change and stasis in the Florida Keys National Marine Sanctuary. *Coral Reefs* 33, 565–577. doi:10.1007/s00338-014-1158-x.
